# Quality of life and its predictors among patients with metastatic cancer in Bangladesh: the APPROACH survey

**DOI:** 10.1186/s12904-023-01301-6

**Published:** 2024-01-03

**Authors:** Rubayat Rahman, Lubna Mariam, Rebecca Su, Chetna Malhotra, Semra Ozdemir

**Affiliations:** 1 Department of Palliative Medicine, Bangabandhu Sheikh Mujib Medical University, Dhaka, Bangladesh; 2 Department of Radiation Oncology, National Institute of Cancer Research & Hospital, Dhaka, Bangladesh; 3 Lien Centre for Palliative Care, Duke-NUS Medical School, 8 College Road, Singapore, 169857 Singapore; 4 Signature Programme in Health Services and System Research, Duke-NUS Medical School, 8 College Road, Singapore, 169857 Singapore

**Keywords:** Metastatic cancer, Palliative care, Quality of life, Bangladesh

## Abstract

**Background:**

This study aimed to assess the health-related quality of life (HRQOL) (physical, functional, emotional, social, spiritual) and psychological (anxiety and depression) well-being and their associations with patient characteristics among patients with metastatic cancer in Bangladesh.

**Methods:**

A convenience sample of 386 Bangladeshi patients with stage IV solid cancers was recruited from a palliative care outpatient department and an inpatient palliative center. Dependent variables included the physical, functional, emotional, social, and overall scores of the Functional Assessment of Cancer Therapy-General (FACT-G) scale, the Functional Assessment of Chronic Illness Therapy-Spiritual Well-being (FACIT-SP) scale, the anxiety, depression, and overall scores of the Hospital Anxiety and Depression (HADS) scale. Linear regressions examined the association between dependent variables and patient characteristics.

**Results:**

A substantial proportion of Bangladeshi patients reported anxiety (59% of outpatients and 55% of inpatients) and depression (60% of outpatients and 73% of inpatients) symptoms. Generally, greater financial difficulty and symptom burden scores were associated with worse health outcomes. Older patients reported poorer functional and spiritual well-being but better anxiety scores. Females reported worse anxiety and depressive symptoms and physical well-being but better spiritual outcomes.

**Conclusions:**

Additional efforts must be directed at improving the HRQOL of patients with metastatic cancer in Bangladesh. Furthermore, assistance should be made more accessible to vulnerable groups, including women, the elderly, and those with financial difficulty.

**Supplementary Information:**

The online version contains supplementary material available at 10.1186/s12904-023-01301-6.

## Background

Cancer poses a significant disease burden worldwide, particularly in low- and middle-income countries (LMICs) [1], where 70% of cancer-related deaths occur [2]. The global cancer burden is expected to increase due to population growth, longer life expectancy, growing urbanization, and lifestyle changes, with LMICs expected to be particularly affected [3]. However, several barriers hinder effective cancer care, including cancer-related shame and stigma, limited knowledge about the disease and accompanying symptoms, and challenges accessing healthcare due to financial constraints and limited resources [4, 5].

In Bangladesh, the focus of this study, 25.9% of the total deaths were attributable to cancer in 2019 [6]. High cancer mortality in the country is generally due to a lack of awareness about the symptoms and risk factors of the disease [7, 8], the stigma associated with a cancer diagnosis in the community [9], inadequate health care [10], and steep co-pay costs without adequate financial support [11, 12]. Cancer patients often experience a poor health-related quality of life (HRQOL) due to discomforting symptoms and adverse treatment-related side effects [13]. Although some Bangladesh-based studies have assessed the HRQOL of patients with advanced cancer [14–17], they have primarily focused on associations between patient HRQOL and cancer type or specific interventions without accounting for patient-level characteristics. None of these studies have assessed the association of socioeconomic and demographic characteristics with HRQOL among patients with metastatic cancer [18].

It is also worth noting that in LMIC including Bangladesh, it is not uncommon for people with advanced disease to lack awareness of their cancer diagnosis due to a tendency to shield patients from potential physical or emotional distress by withholding cancer diagnosis [19]. While direct evidence from Bangladesh is lacking, studies conducted with people originating from Bangladesh and other Asian societies have demonstrated that cancer disclosure is often viewed as undesirable [20–22].

Bangladesh has a comprehensive tax-funded health system on paper; however, its utilization and effectiveness fall short in providing adequate health coverage without imposing undue financial burden [23, 24]. In addition, the health sector has not received sufficient priority in national development policies, leading to a lack of sustained improvements. As a result, both the allocation and the level of government spending on healthcare in Bangladesh are relatively low, accounting for only 5.4% of the overall government budget and 0.95% of GDP [23].

This study sought to assess the HRQOL of Bangladeshi patients with advanced cancer, considering their general well-being (physical, functional, emotional, and social/family), as well as spiritual and psychological well-being in order to have a comprehensive understanding of their suffering from cancer [25, 26]. The aim of this study was to better understand the role various socio-economic and demographic factors play in variations in HRQOL outcomes. Building upon evidence from studies conducted in other LMICs, we hypothesized that female patients [9, 27, 28], older individuals [29, 30], those facing financial difficulty [28, 31], those with lower social support such as unmarried individuals [30, 32], and patients with lower levels of education [33] would report worse HRQOL outcomes compared to their counterparts.

## Methods

### Design and settings

A cross-sectional survey was conducted as part of the “Asian Patient Perspectives Regarding Oncology Awareness, Care and Health (APPROACH) project across nine LMICs in Asia. This larger project set out to investigate patients living with advanced cancer regarding various domains: HRQOL [33], quality of care [34], perceived and preferred levels of involvement in decision-making [35], understanding of prognosis [36], awareness and utilization of hospice palliative care services [37], mental health services [38], as well as cancer-related self-blame [39] and social stigma [40].

As one of the participating APPROACH study sites, the survey was conducted in collaboration with the local investigators in two study sites: (1) Dhaka, Bangladesh: an outpatient palliative care department in the National Institute of Cancer Research and Hospital (NICRH) and (2) an inpatient palliative care center located in Bangabandhu Sheikh Mujib Medical University (BSMMU). The NICRH is the only specialized government institute and tertiary-level cancer hospital in Bangladesh while BSMMU is the first and only public medical university in Bangladesh. For the purpose of this study, patients from NICRH were referred to as “outpatients”, while those from BSMMU were referred to as “inpatients”.

### Ethics

The study was performed in line with the principles of the Declaration of Helsinki and approved by the Institutional Review Board of the National University of Singapore (reference: B-15-319), as well as those of the participating institutions in Bangladesh, the NICRH (reference: NICRH/ Ethics/ 2016/213) and the Centre for Palliative Care at BSMMU (reference: BSMMU/2016/5171).

### Participants

We targeted to enroll a convenience sample of approximately 200 patients at each site who met the following inclusion criteria: being a Bangladeshi citizen, aged ≥ 21 years, diagnosed with stage IV solid cancer, being aware of cancer diagnosis, and willingness to provide consent.

Eligible patients identified by the site principal investigator or treating physicians, were referred to the study coordinator, or by the study coordinator reviewing medical records. Patients were recruited between April and November 2018 at the outpatient clinic and between February 2017 and October 2019 at the inpatient center. All participants provided written consent.

### Survey design and development

The survey questionnaire included validated scales and questions developed by the study investigators. Questions (developed by the study investigators) were written in English and professionally translated into Bangla, which were then back translated to English. All discrepancies were reconciled. Supplementary Material provides the survey questions used in the study. The details of the validated scales are provided below.

### Health-related quality of life outcomes

We assessed patients’ HRQOL using the validated 27-item Functional Assessment of Cancer Therapy-General (FACT-G) scale [41, 42]. The questionnaire covers four domains for health-related quality of life, including physical (FACT-GP), functional (FACT-GF), emotional (FACT-GE), and social/ family (FACT-GS) well-being. We summed scores from each domain to obtain a total score (range: 0–108), where higher scores indicate greater well-being.

We assessed patients’ spiritual well-being using the Functional Assessment of Chronic Illness Therapy-Spiritual Well-being scale (FACIT-Sp) [43]. The scores in the FACIT-Sp scale can range from 0 to 48, with higher scores indicating greater spiritual well-being. We also included the Hospital Anxiety and Depression Scale (HADS) to assess patients’ psychological distress levels [44]. The two HADS subscales: Anxiety (HADS-A) and Depression (HADS-D) with 7 questions each assessing anxiety and depression levels, respectively. Scores can range from 0 to 21 for both anxiety and depression, with higher scores indicating greater psychological distress. Altogether, the total HADS score can range from 0 to 42. A score between 0 and 7 for either subscale is regarded as the normal range, a score between 8 and 10 indicates a probable borderline psychological distress state, and a score of 11 or more indicates a probable mood disorder.

While licensed Bengali FACT-G and HADS translations were used, we translated the FACIT-Sp based on suggestions provided by the site investigators. The translation procedures and pilot testing for the FACIT-Sp scale followed the guidance provided by FACIT, and the final version was approved by the FACIT license owners.

Acceptable internal consistency of the FACT-G, FACIT-Sp, and HADS scales were established in this study population with a Cronbach’s alpha of 0.75 for the FACT-G, 0.79 for the FACT-Sp, and 0.84 for the HADS [45].

### Patient characteristics

The survey also included questions on participants’ socio-demographic characteristics (e.g. age, gender, years of education, and marital status). We evaluated patients’ financial status by summing the response scores for the following three questions:

How well would their financial situation enable them to:

1) cover the cost of their treatment,

2) take care of their daily needs, and.

3) buy those little ‘extras’ or small luxuries?

The resulting total score can range from 3 to 9, with 3 representing least financial difficulty and 9 the highest. These questions were previously used and tested with cancer and heart patients [31, 46].

We measured symptom burden by summing responses across ten symptoms (pain, shortness of breath, constipation, weight loss, vomiting, swelling, mouth and throat dryness, lack of energy, nausea, and any other symptom) that patients with cancer commonly experience. The list of symptoms was taken from FACIT-PAL-14 [47]. The resulting score ranged from 10 to 50, with 10 representing the least burden and 50 the highest [48].

### Statistical analysis

We presented descriptive statistics for socio-demographic characteristics and HRQOL outcomes for the two sites. We compared the FACT-G, FACIT-Sp, and HADS scores between the two sites. We compared the means between samples using t-tests and also calculated Cohen’s D effect sizes. Effect sizes of 0.8 and above were considered large [49].

We used linear regressions to estimate the association between patient characteristics and HRQOL outcomes. We fitted nine separate models for each HRQOL outcome as the dependent variable - overall FACT-G, FACT-GP, FACT-GF, FACT-GE, FACT-GS, FACIT-Sp, HADS, HADS-A, and HADS-D.

The predictors or independent variables included age, years of education, financial difficulty score, symptom burden, gender (female = 1, male = 0), and marital status (unmarried/separated/divorced/widowed/never married) = 1, otherwise = 0). In all models, the site (0 = patients from the outpatient clinic, 1 = patients from the inpatient center) variable was used to control for any unobserved differences between the two hospitals.

All analyses were conducted in STATA 15 by a data analyst trained in econometrics.

## Results

### Study sample

The mean ages of the 386 patients enrolled in the study were 49.6 ± 13.6 for the outpatients and 48.1 ± 13.8 for the inpatients (Table 1). There were significantly more females enrolled at the outpatient clinic than at the inpatient center (51% vs. 24%; p-value < 0.001). Across both sites, most patients (80–88%) were married. Inpatients had significantly more years of education than outpatients (8.8 ± 6.6 versus 5.1 ± 5.9; p-value < 0.001). The mean financial difficulty scores were similar at both sites (8.1 ± 1.5 versus 8.3 ± 1.3; p-value = 0.134). However, inpatients had significantly greater symptom burden than outpatients (18.9 ± 4.9 versus 14.2 ± 5.7; p-value < 0.001).

**Table 1:** Patient Characteristics

Mean (SD) / N (%)			
Variable	NICRH (outpatient department) (N = 189 )	BSMMU (inpatient center) (N = 197 )	P-Value^1,2^
Age	49.6 (13.6)	48.1 (13.8)	0.282
Education	5.1 (5.9)	8.8 (6.6)	< 0.001
Financial Difficulty (3 to 9)	8.3 (1.3)	8.1 (1.5)	0.134
Symptom Burden (0 to 40)	14.2 (5.7)	18.9 (4.9)	< 0.001
**Gender**			
Male	93 (49%)	150 (76%)	< 0.001
Female	96 (51%)	47 (24%)	< 0.001
**Marital status**			
Not Married	37 (20%)	24 (12%)	0.047
Married	152 (80%)	173 (88%)	0.047

^*1*^ *Two-sample t test using groups was used to compare means of categorical variables from site sociodemographic data and determine P-value*

^*2*^ *Chi^2 was used to compare means of continuous variables from site sociodemographic data and determine P-value*

Inpatients reported significantly lower mean HRQOL (overall FACT-G score) than outpatients (46.4 ± 10.7 versus 53.0 ± 13.2; p-value < 0.001) (Table 2). Specifically, inpatients reported significantly lower physical well-being (10.2 ± 3.6 versus 13.7 ± 4.8; p-value < 0.001), functional well-being (8.6 ± 3.9 versus 10.2 ± 5.5; p-value = 0.001), emotional well-being (9.3 ± 4.9 versus 10.3 ± 4.7; p-value = 0.035), and spiritual well-being (22.4 ± 6.2 versus 29.9 ± 6.5; p-value < 0.001). Effect sizes for the magnitude of difference between the two sites were large (i.e., over 0.8) for spiritual well-being (1.18) and physical well-being (0.82).

**Table 2:** Patient-reported HRQOL outcomes (NICRH: outpatient department) versus (BSSMU: inpatient center)

Mean (SD)				
Variables	NICRH (outpatient department) (N = 189 )	BSMMU (inpatient center) (N = 197 )	p-value	Cohen’s D^1^ (95% CI)
FACT-G (Overall) (out of 108)	53.0 (13.2)	46.4 (10.7)	< 0.001	-0.55 (-0.76, -0.35)
FACT-G Physical well-being (FACT-GP) (out of 28)	13.7 (4.8)	10.2 (3.6)	< 0.001	-0.82 ( -1.03, -0.62)
FACT-G Functional well-being (FACT-GF) (out of 28)	10.2 (5.5)	8.6 (3.9)	0.001	-0.33 (-0.54, -0.13)
FACT-G Emotional well-being subscale (FACT-GE) (out of 28)	10.3 (4.7)	9.3 (4.9)	0.035	-0.22 (-0.42, -0.01)
FACT-G Social well-being subscale (FACT-GS) (out of 28)	18.9 (5.2)	18.3 (4.7)	0.279	-0.11 (-0.31, 0.09)
FACIT-Sp (out of 48)	29.9 (6.5)	22.4 (6.2)	< 0.001	-1.18 (-1.39, -0.96)
HADS (Overall) (out of 42)	22.0 (5.6)	22.9 (5.3)	0.088	0.17 ( -0.03, 0.37)
HADS Anxiety (HADS-A) (out of 21)	10.9 (3.8)	10.5 (3.3)	0.333	-0.10 (-0.30, 0.10)
0–7, n (%)	36 (19.05)	31 (15.74)		
8 − 1, n (%)	42 (22.22)	58 (29.44)		
11 and above, n (%)	111 (58.73)	108 (54.82)	0.245	
HADS Depression (HADS-D) (out of 21)	11.1 (3.1)	12.4 (3.0)	< 0.001	0.43 (0.23, 0.63)
0–7, n (%)	16 (8.47)	7 (3.55)		
8–10, n (%)	59 (31.22)	46 (23.35)		
11 and above, n (%)	114 (60.32)	144 (73.10)	< 0.05	

^*1*^ *Cohen’s D effect sizes 0.8 and over will be considered large*

HADS overall scores and anxiety scores were not significantly different between the two sites, but inpatients reported higher depressive symptoms compared to outpatients (12.4 ± 3.0 versus 11.1 ± 3.1; p-value < 0.001). 59% of the outpatients and 55% of the inpatients reported an anxiety score of at least 11 (p = 0.245) while 60% of the outpatients and 73% of the inpatients reported a depression score of at least 11 (p-value < 0.05), indicative of probable mood disorder for these patients.

### Associations of patient characteristics with HRQOL outcomes

Older age was associated with poorer functional (β= -0.037, p-value < 0.05) well-being but also with lower anxiety scores (β= -0.040, p-value < 0.01) (Table 3). Patients with higher financial difficulty scores reported poorer functional (β= -0.781, p-value < 0.01), emotional (β= -0.572, p-value < 0.01), social (β= -0.622, p-value < 0.01), and spiritual well-being (β= -1.434, p-value < 0.01).

**Table 3:** Linear regressions of HRQOL outcomes on patient characteristics

	(1)	(2)	(3)	(4)	(5)	(6)	(7)	(8)	(9)
	**FACT-G**	**FACT-G-PWB**	**FACT-G-FWB**	**FACT-G-EWB**	**FACT-G-SWB**	**FACI-Sp**	**HADS Total**	**HADS Anxiety**	**HADS Depression**
Age	-0.018	0.005	-0.037^**^	-0.002	0.026	-0.044^*^	-0.047^**^	-0.040^***^	-0.007
	(-0.43)^1^	(0.36)	(-2.03)	(-0.09)	(1.32)	(-1.85)	(-2.27)	(-2.94)	(-0.59)
Years of Education	0.049	-0.032	0.005	0.026	0.059	0.007	0.046	0.036	0.010
	(0.54)	(-1.14)	(0.13)	(0.65)	(1.40)	(0.14)	(1.02)	(1.22)	(0.39)
Financial Difficulty Score	-2.262^***^	-0.243^*^	-0.781^***^	-0.572^***^	-0.622^***^	-1.434^***^	0.164	0.112	0.052
	(-5.44)	(-1.89)	(-4.38)	(-3.12)	(-3.25)	(-6.14)	(0.81)	(0.83)	(0.45)
Symptom Burden	-0.895^***^	-0.492^***^	-0.192^***^	-0.163^***^	-0.056	-0.077	0.254^***^	0.130^***^	0.125^***^
	(-8.77)	(-15.62)	(-4.38)	(-3.61)	(-1.19)	(-1.34)	(5.10)	(3.94)	(4.39)
Female	-1.382	-0.706^*^	-0.210	-0.763	0.289	1.779^**^	1.634^***^	1.035^***^	0.599^*^
	(-1.13)	(-1.86)	(-0.40)	(-1.41)	(0.51)	(2.58)	(2.73)	(2.62)	(1.75)
Unmarried	0.541	0.570	0.571	-0.649	0.025	0.001	0.140	0.225	-0.084
	(0.36)	(1.22)	(0.88)	(-0.98)	(0.04)	(0.00)	(0.19)	(0.46)	(-0.20)
Inpatient center	-3.459^***^	-1.229^***^	-0.952^*^	-0.758	-0.519	-7.034^***^	0.001	-0.829^**^	0.830^**^
	(-2.72)	(-3.13)	(-1.75)	(-1.35)	(-0.89)	(-9.85)	(0.00)	(-2.02)	(2.34)
Constant	85.76^***^	22.87^***^	21.22^***^	17.85^***^	23.08^***^	44.08^***^	18.26^***^	9.343^***^	8.916^***^
	(17.16)	(14.82)	(9.90)	(8.06)	(10.04)	(15.70)	(7.48)	(5.81)	(6.41)
Observations	386	386	386	384	386	386	386	386	386

^1^*t* statistics in parentheses

^*^p < 0.10, ^**^p < 0.05, ^***^p < 0.01

Similarly, patients with higher symptom burden reported poorer physical (β= -0.492, p-value < 0.01), functional (β= -0.192, p-value < 0.01), and emotional well-being (β= -0.163, p-value < 0.01), and greater anxiety (β = 0.130, p-value < 0.01) and depressive scores (β = 0.125, p-value < 0.01). Compared to males, female patients reported slightly greater anxiety scores (β = 1.035, p-value < 0.01) but reported better spiritual well-being (β = 1.779, p-value < 0.05).

Years of education and marital status were not significant predictors of HRQOL outcomes in our patient sample.

## Discussion

### Patient quality of life

In line with past research conducted in Bangladesh [14, 50, 51], patients with metastatic cancer reported low HRQOL outcomes in terms of physical, functional, emotional, social, and spiritual well-being. These results might be due to Bangladesh’s limited treatment and palliative-care resources as well as limited medical infrastructure, educated workforce, and service availability (e.g., radiotherapy units, radiation treatment) [52–54]. While there are approximately twenty hospitals that offer cancer care in Bangladesh, most cancer patients are referred to an already-overwhelmed public healthcare system, where the number of patients greatly outstrips the number of inpatient beds [52].

Furthermore, patients from both sites reported poor psychological well-being. About half of all patients in this study reported scores indicative of probable anxiety, while a higher proportion reported scores indicative of probable depression. These results suggest there is a need for routine screening for anxiety and depression in the inpatient and outpatient setting, and further investigation for those found to have higher scores. These findings are comparable to those found in other Asian countries [38, 55] and notably higher than those reported in a recent meta-analysis encompassing research conducted with samples from high-income or upper-middle-income countries [56]. Nevertheless, these findings raise concerns and underscore the need to improve psychosocial support for patients with metastatic cancer in Bangladesh, as most country hospitals have limited resources for managing patients’ psychological and emotional difficulties [57]. There is a scarcity of healthcare workers trained in providing mental health services, and the number of psychiatrists available is extremely limited (0.16 per 100,000 population) [58]. Additionally, mental health programs receive inadequate funding, with mental health expenditures accounting for only 0.44% of the total health budget [58]. These resources need to be expanded in order to improve mental well-being among patients with metastatic cancer.

Patients recruited at the inpatient palliative care center reported worse HRQOL than patients from the outpatient palliative care clinic. We posit that these differences are unlikely to be attributed to differences in care but rather due to the fact that patients recruited at the inpatient center are likely to be much sicker and nearing the end of their lives compared to patients recruited at the outpatient clinic. In support of this, our study found that symptom burden scores for inpatients were significantly higher than those for outpatients. The largest difference between the two sites was seen in their spiritual well-being scores, with inpatients reporting markedly low scores indicative of reduced spiritual status as they approached the end of their lives. These findings highlight the need for improving awareness and capacity, such as expanding healthcare manpower and resources [59], opioid availability [60], and hospice care services and awareness of these services [37] which are severely lacking or limited in the country.

### Associations between HRQOL and patient characteristics

Symptom burden was the most common predictor of lower HRQOL in all domains, except for social and spiritual well-being. Symptom burden was also a significant predictor of higher anxiety and depression scores. These results are consistent with past studies, which have shown that symptoms, such as pain [61], fatigue [61], weight loss [62], and nausea and vomiting [63], affect patients’ physical and functional well-being and are associated with worse emotional and psychological well-being.

Consistent with our hypotheses, our results show that greater financial difficulty was associated with poorer functional, emotional, social, and spiritual well-being. Financial difficulty among cancer patients is common, disproportionately affecting younger and socioeconomically disadvantaged patients and associated with worse HRQOL [28, 31, 64]. People with higher financial difficulty scores may be suffering from emotional problems due to their concerns about their finances [31, 65, 66]. Financial difficulty may stem from a cancer-related loss of family income, social discrimination, and rejection, which are all associated with worse HRQOL [66]. In addition, greater financial difficulty may also arise from increased use of health care services and higher medical expenses which, in return, indicate worse patient HRQOL. Overall, people with higher financial difficulty scores may suffer from emotional problems and could have poorer physical and functional well-being scores.

Our study also showed that older age was associated with poorer functional well-being, which is unsurprising as older patients are more likely to experience functional problems. Conversely, older age was associated with lower anxiety [67], since older patients are more likely to accept the outcomes of their cancer compared to younger patients who, otherwise, would have expected to live a longer and healthier life [68].

Compared to male patients, female patients reported better spiritual well-being but worse anxiety. A worse anxiety may be due to worries regarding their fertility and responsibilities towards family, especially their children [69]. On the other hand, female patients may be better at finding faith and meaning in what they are going through compared to male patients [70]. It may also be that patients who experience worse anxiety are more likely to seek spiritual outlets and thereby improve their spiritual well-being [71].

### Limitations

Despite the importance of our findings, several limitations in our research should be highlighted. First, the results may not be nationally representative or apply to other LMICs since we recruited patients only from two specific sites. This limited sample size and site selection may restrict the wider applicability of our findings. Second, we only included patients who were aware of their cancer diagnosis, potentially excluding a subset of individuals who may be unaware of their condition due to the reasons previously mentioned. Third, the study sites did not systematically track and record detailed information about the recruitment process, such as the number of individuals screened, eligible, and approached for participations. This lack of information may affect the transparency and completeness of the recruitment process. Fourth, our study design was cross-sectional, which means that we cannot draw causal conclusions. Lastly, the mean age of the patients in this study is lower compared to similar studies in the region [35], but it aligns with other cancer studies within the country [72, 73]. In LMICs, the occurrence of cancer in young age is often attributed to factors such as lower life expectancy and a relatively younger population structure. However, the specific reasons for this trend among Bangladeshi patients remain unclear [72].

### Implications

The findings of this study provide a better understanding of the HRQOL experienced by metastatic cancer patients recruited from palliative care units in Bangladesh and help in predicting which groups are likely to experience poorer HRQOL. We hope that the identification of factors predicting poor HRQOL will enable the development of more targeted approaches for care delivery and for informing cancer and palliative centers in Bangladesh and other low- and middle-income countries. Importantly, our findings highlight the need for expanding healthcare services offered to cancer patients, training clinicians in palliative care, and integrating palliative care infrastructure into the mainstream healthcare system to increase the HRQOL of Bangladeshi patients with metastatic cancer.

## Conclusion

By examining the HRQOL and its socio-economic and demographic predictors among patients with metastatic cancer in Bangladesh, this study highlighted several important findings. First, patients reported low HRQOL across various domains, including physical, functional, emotional, social, and spiritual well-being. They also reported high levels of anxiety and depression. The levels of HRQOL were even worse for patients recruited from the inpatient clinic. Second, higher financial and symptom burdens were significantly associated with poorer HRQOL outcomes in general. Being female, younger, and having a higher symptom burden were associated with higher anxiety and depression. Our results highlight the critical need to improve funding for palliative care, symptom management, and mental health services specifically targeted towards patients with metastatic cancer in lower-middle-income countries such as Bangladesh.

### Electronic supplementary material

Below is the link to the electronic supplementary material.

Supplementary Material 1: Survey Questions Used in the Paper

## Data Availability

The datasets used and/or analyzed during the current study are available from the corresponding author on reasonable request.
