# Supplementary material for: Quality of life and its predictors among patients with metastatic cancer in Bangladesh: the APPROACH survey
Source: BMC Palliat Care. 2024 Jan 3;23:2. doi: 10.1186/s12904-023-01301-6 (PMC10762837; doi:10.1186/s12904-023-01301-6)
Supplement: Supplementary file 1 — Supplementary Material 1: Survey Questions Used in the Paper [file 12904_2023_1301_MOESM1_ESM.docx]

**Supplementery Material**

**Patient Recruitment**

At the outpatient clinic, 11 patients were found to be ineligible: five of them were below the minimum age, while the remaining six patients were not diagnosed with solid cancer. At the inpatient center, three patients were unable to participate: two of them were below the minimum age, while one patient was not diagnosed with solid cancer.

**Survey Questions used in the Paper**

| Asian Patient Perspectives Regarding Oncology Awareness, Care, and Health (APPROACH) | | | | | | |  |
| --- | --- | --- | --- | --- | --- | --- | --- |
| For interviewer: Fill in the following information after you have completed the questionnaire. | | | | | | | |
| Participant Code:  (from consent form) | |  | Date Of Interview (dd/mm/yyyy): | | | ___/___/_____ | |
| Time Started: |  | Time Ended: | |  | Total Interview Time: | | |
| Interviewer Name: | | | |  | |  | |
| Country: |  | | | interview language: | |  | |
| Name of the institution: | | | | | |  | |
| Patient type: □1 Outpatient □2 Inpatient | | | | | | | |
| Site of recruitment:  □1 Department of Medical Oncology  □7 Department of Radiation Oncology  □8 Palliative Care Unit  □9 Other | | | | | | | |
| Fill in the information from patient’s medical records. | | Patient Gender: □1 Male □2 Female | | | | | |
|  |  | Patient’s Date of Birth (dd/mm/yyyy):____/_____/_____ | | | | | |
|  |  | Type of Cancer: _____________________________ | | | | | |

**INTRODUCTION**

We are conducting a survey to understand the quality of life of patients, quality of care they are currently receiving and their treatment preferences. Your opinions are important to the success of this study. The survey usually takes about **45 minutes**.

There are no right or wrong answers to the questions and you do not have to respond to any questions that you feel uncomfortable answering. Your identity and the information given will be kept strictly confidential and only group data will be reported.

**SECTION S: SCREENER**

| **S1** | **Have you ever been diagnosed with any of the following health conditions? Check all that apply.** | | |
| --- | --- | --- | --- |
|  |  | **Health Conditions** | **Yes** |
|  | □1 | Diabetes | □ |
|  | □2 | Heart conditions (e.g. heart attack, blocked blood vessels) | □ |
|  | □3 | Lung/Liver disease (e.g. bronchitis, hepatitis) | □ |
|  | □4 | Cancer | □ |
| **[TERMINATE if option 4 ‘Cancer’ is NOT checked]**  **[Thank you for your interest, but you are not eligible to continue with this survey]** | | | |

Refuse to answer

**SECTION A: BASIC INFORMATION**

**[For questions A1 to A5, you do not need to read the response choices out loud to the patient. Allow the patient to first respond directly, and prompt him/her with relevant choices depending upon his/her response]**

| **A1**  88888 | **What is your age?**  **years old** | | | | | |
| --- | --- | --- | --- | --- | --- | --- |
| **A2**  888 | **How many years of education have you completed (including higher education)?**  **years** | | | | | |
| **A3**  888 | **What is your current marital status?** | | | | | |
|  | □1 | Married | | □4 | Divorced | |
|  | □2 | Separated | | □5 | Never married | |
|  | □3 | Widowed | |  |  | |
| **A4**  888 | **What is your religion?** | | | | | |
|  | □1 | Hindu | | □7 | | Jewish |
|  | □2 | Muslim | | □8 | | Parsi/Zoroastrian |
|  | □3 | Christian (including Roman Catholic, Protestant, Orthodox, other) | | □9 | | Taoist |
|  | □4 | Sikh | | □10 | | Confucian |
|  | □5 | Buddhist | | □11 | | No religion |
|  | □6 | Jain | | □12 | | Other, specify: ______________ |
| **A4.2**  888 | **Which division are you from?** | | | | | |
|  | □1 | Barisal Division | | □5 | | Mymensingh Division |
|  | □2 | Chittagong Division | | □6 | | Rajshahi Division |
|  | □3 | Dhaka Division | | □7 | | Rangpur Division |
|  | □4 | Khulna Division | | □8 | | Sylhet Division |
| **A5**  88888 | **What type of cancer have you been diagnosed with?**   \| □1 \| Bladder \| □11 \| Lung \| \| --- \| --- \| --- \| --- \| \| □2 \| Brain \| □12 \| Nasopharyngeal \| \| □3 \| Breast \| □13 \| Oesophageal \| \| □4 \| Cervical \| □14 \| Ovarian \| \| □5 \| Colorectal \| □15 \| Oral \| \| □6 \| Endometrial \| □16 \| Pancreatic \| \| □7 \| Gastric \| □17 \| Prostate \| \| □8 \| Intestinal \| □18 \| Vulva \| \| □9 \| Kidney \| □19 \| Others, please specify _______ \| \| □10 \| Liver \| □20 \| Don’t know \| | | | | | |
| **A6**  88888 | **Do you know the current stage (i.e. severity) of your cancer?** | | | | | |
|  | □1 | | Early Stage (Stage I, II or III) | | | |
|  | □2 | | Advanced Stage (Stage IV) | | | |
|  | □3 | | I don’t know | | | |
| **A7**  88888 | **[If A6=2] What was the stage (i.e. severity) of your cancer when you first learned about it?** | | | | | |
|  | □1 | | Early Stage (Stage I, II or III) | | | |
|  | □2 | | Advanced Stage (Stage IV) | | | |
|  | □3 | | I don’t know | | | |

Refuse to answer

**SECTION B: QUALITY OF LIFE**

Refuse to answer

| **B1^[[1]](#footnote-1)^** | **Below is a list of statements that other people with your illness have said are important or bothersome for them. Some of the items may appear to be repetitive, but please bear with us. Please indicate one number per line as it applies to the past 7 days.** | | | | | | | |
| --- | --- | --- | --- | --- | --- | --- | --- | --- |
|  |  |  | | **Not at all** | **A little bit** | **Some-what** | **Quite a bit** | **Very much** |
| 88888 | B1,1 GP3 | Because of my physical condition, I have trouble meeting the needs of my family | | 0 | 1 | 2 | 3 | 4 |
| 88888 | B1.2 GP5 | I am bothered by side effects of treatment | | 0 | 1 | 2 | 3 | 4 |
| 88888 | B1.3 GP6 | I feel ill | | 0 | 1 | 2 | 3 | 4 |
| 88888 | B1.4 GP7 | I am forced to spend time in bed | | 0 | 1 | 2 | 3 | 4 |
|  | | | | | | | | |
|  |  |  | | **Not at all** | **A little bit** | **Some-what** | **Quite a bit** | **Very much** |
| 88888 | B1.5 GS1 | I feel close to my friends | | 0 | 1 | 2 | 3 | 4 |
| 88888 | B1.6 GS2 | I get emotional support from my family | | 0 | 1 | 2 | 3 | 4 |
| 88888 | B1.7 GS3 | I get support from my friends | | 0 | 1 | 2 | 3 | 4 |
| 88888 | B1.8 GS4 | My family has accepted my illness | | 0 | 1 | 2 | 3 | 4 |
| 88888 | B1.9 GS5 | I am satisfied with family communication about my illness | | 0 | 1 | 2 | 3 | 4 |
| 88888 | B1.10 GS6 | I feel close to my partner (or the person who is my main support) | | 0 | 1 | 2 | 3 | 4 |
|  |  | *Regardless of your current level of sexual activity, please answer the following question. If you prefer not to answer it, please mark this box and go to the next question.* | | | | | | |
|  | B1.11 GS7 | I am satisfied with my sex life | | 0 | 1 | 2 | 3 | 4 |
|  | | | | | | | | |
|  |  |  | | **Not at all** | **A little bit** | **Some-what** | **Quite a bit** | **Very much** |
| 88888 | B1.12GE1 | I feel sad | | 0 | 1 | 2 | 3 | 4 |
|  |  |  | | **Not at all** | **A little bit** | **Some-what** | **Quite a bit** | **Very much** |
| 88888  Refuse to answer | B1.13 GE2 | I am satisfied with how I am coping with my illness | | 0 | 1 | 2 | 3 | 4 |
| 88888 | B1.14 GE3 | I am losing hope in the fight against my illness | | 0 | 1 | 2 | 3 | 4 |
| 88888 | B1.15 GE4 | I feel nervous | | 0 | 1 | 2 | 3 | 4 |
| 88888 | B1.16 GE5 | I worry about dying | | 0 | 1 | 2 | 3 | 4 |
| 88888 | B1.17 GE6 | I worry that my condition will get worse | | 0 | 1 | 2 | 3 | 4 |
|  | | | | | | | | |
|  |  |  | | **Not at all** | **A little bit** | **Some-what** | **Quite a bit** | **Very much** |
| 88888 | B1.18 GF1 | I am able to work (include work at home) | | 0 | 1 | 2 | 3 | 4 |
| 88888 | B1.19 GF2 | My work (include work at home) is fulfilling | | 0 | 1 | 2 | 3 | 4 |
| 88888 | B1.20 GF3 | I am able to enjoy life | | 0 | 1 | 2 | 3 | 4 |
| 88888 | B1.21 GF4 | I have accepted my illness | | 0 | 1 | 2 | 3 | 4 |
| 88888 | B1.22 GF5 | I am sleeping well | | 0 | 1 | 2 | 3 | 4 |
| 88888 | B1.23 GF6 | I am enjoying the things I usually do for fun | | 0 | 1 | 2 | 3 | 4 |
| 88888 | B1.24GF7 | I am content with the quality of my life right now | | 0 | 1 | 2 | 3 | 4 |
|  | | | | | | | | |
|  |  | |  | **Not at all** | **A little bit** | **Some-what** | **Quite a bit** | **Very much** |
| 88888 | B1.25 Sp1 | | I feel peaceful | 0 | 1 | 2 | 3 | 4 |
| 88888 | B1.26 Sp2 | | I have a reason for living | 0 | 1 | 2 | 3 | 4 |
| 88888 | B1.27 Sp3 | | My life has been productive | 0 | 1 | 2 | 3 | 4 |
| 88888 | B1.28 Sp4 | | I have trouble feeling peace of mind | 0 | 1 | 2 | 3 | 4 |
| 88888 | B1.29 Sp5 | | I feel a sense of purpose in my life | 0 | 1 | 2 | 3 | 4 |
| Refuse to answer |  | |  | **Not at all** | **A little bit** | **Some-what** | **Quite a bit** | **Very much** |
| 88888 | B1.30 Sp6 | | I am able to reach down deep into myself for comfort | 0 | 1 | 2 | 3 | 4 |
| 88888 | B1.31 Sp7 | | I feel a sense of harmony within myself | 0 | 1 | 2 | 3 | 4 |
| 88888 | B1.32 Sp8 | | My life lacks meaning and purpose | 0 | 1 | 2 | 3 | 4 |
| 88888 | B1.33 Sp9 | | I find comfort in my faith or spiritual beliefs | 0 | 1 | 2 | 3 | 4 |
| 88888 | B1.34 Sp10 | | I find strength in my faith or spiritual beliefs | 0 | 1 | 2 | 3 | 4 |
| 88888 | B1.35 Sp11NI | | Difficult times have strengthened my faith or spiritual beliefs | 0 | 1 | 2 | 3 | 4 |
| 88888 | B1.36 Sp12NI | | Even during difficult times, I know that things will be okay | 0 | 1 | 2 | 3 | 4 |
| 88888 | B1.37 Sp21 | | I feel hopeful | 0 | 1 | 2 | 3 | 4 |

The next three questions will focus on your finances.

| **B2**  88888 | **How well does the amount of money you have enable you to cover the cost of your treatment?** | | |
| --- | --- | --- | --- |
|  | □1 | Very well | |
|  | □2 | Fairly well | |
|  | □3 | Poorly | |
| **B3**  88888 | **How well does the amount of money you have take care of your daily needs?** | | |
|  | □1 | | Very well |
|  | □2 | | Fairly well |
|  | □3 | | Poorly |
| **B4**  88888 | **How well does the amount of money you have enable you to buy those little ‘extras’, that is, those small luxuries?** | | |
|  | □1 | | Very well |
|  | □2 | | Fairly well |
|  | □3 | | Poorly |

Refuse to answer

| **B13^[[2]](#footnote-2)^** | **Below is a list of symptoms that people with your illness commonly experience. Please circle or mark one number per line to indicate your response as it applies to the past 7 days.**  Refuse to answer | | | | | | | | |
| --- | --- | --- | --- | --- | --- | --- | --- | --- | --- |
|  |  | **SYMPTOM MANAGEMENT** | | | **Not at all** | **A little bit** | **Some-what** | **Quite a bit** | **Very much** |
| 88888 | B13.1 GP4 | I have pain | | | 0 | 1 | 2 | 3 | 4 |
| 88888 | B13.2  B1 | I have been short of breath | | | 0 | 1 | 2 | 3 | 4 |
| 88888 | B13.3  PAL5 | I am constipated | | | 0 | 1 | 2 | 3 | 4 |
| 88888 | B13.4  C2 | I am losing weight | | | 0 | 1 | 2 | 3 | 4 |
| 88888 | B13.5  O2 | I have been vomiting | | | 0 | 1 | 2 | 3 | 4 |
| 88888 | B13.6  PAL6 | I have swelling in parts of my body | | | 0 | 1 | 2 | 3 | 4 |
| 88888 | B13.7  PAL7 | My mouth and throat are dry | | | 0 | 1 | 2 | 3 | 4 |
| 88888 | B13.8  GP1 | I have lack of energy | | | 0 | 1 | 2 | 3 | 4 |
| 88888 | B13.9  GP2 | I have nausea | | | 0 | 1 | 2 | 3 | 4 |
| 88888 | B13.10 | Any other symptom, please specify:  ________________ | | |  | 1 | 2 | 3 | 4 |
| **B14**  88888 | **(If any of B13.1 to B13.10 ≠0) Did you tell your doctor about your symptoms?** | | | | | | | | |
|  | □1 | | | Yes | | | | | |
|  | □2 | | | No | | | | | |
| **B15**  88888 | **(If B14=2) Why did you not tell your doctor about your symptoms? Check all that apply.** | | | | | | | | |
|  | □1 | | I can manage my symptoms myself | | | | | | |
|  | □2 | | I want the doctor to focus on the treatment of my cancer | | | | | | |
|  | □3 | | I did not get an opportunity to tell the doctor | | | | | | |
|  | □4 | | I believe that it is easier to put up with symptoms than with the side effects that come from medicines to treat these symptoms. | | | | | | |
|  | □5 | | I will use medicines only as a last resort to treat my symptoms | | | | | | |
|  | □6 | | I believe that good patients avoid talking about symptom/s | | | | | | |
|  | □7 | | I can get addicted easily to medicines needed to treat these symptoms | | | | | | |
|  | □8 | | I believe that tolerating symptoms builds character—it’s good for me. | | | | | | |
|  | □9 | | Any other reason, specify________________________________________ | | | | | | |

Refuse to answer

| **B16^[[3]](#footnote-3)^** | **The following questions will help us to know how you are feeling. For each of the following questions, please indicate how you have been feeling in the PAST WEEK. You do not have to think too much to answer. Your immediate response is the best.** | | |
| --- | --- | --- | --- |
| 88888 | **B16.1** | **I feel tense or wound up.** | |
|  |  | □1 | Most of the time |
|  |  | □2 | A lot of the time |
|  |  | □3 | From time to time, occasionally |
|  |  | □4 | Not at all |
| 88888 | **B16.2** | **I still enjoy the things I used to enjoy.** | |
|  |  | □1 | Definitely as much |
|  |  | □2 | Not quite so much |
|  |  | □3 | Only a little |
|  |  | □4 | Hardly at all |
| 88888 | **B16.3** | **I get a sort of frightened feeling as if something awful is about to happen.** | |
|  |  | □1 | Very definitely and quite badly |
|  |  | □2 | Yes, but not too badly |
|  |  | □3 | A little, but it doesn’t worry me |
|  |  | □4 | Not at all |
| 88888 | **B16.4** | **I can laugh and see the funny side of things.** | |
|  |  | □1 | As much as I always could |
|  |  | □2 | Not quite so much now |
|  |  | □3 | Definitely not as much now |
|  |  | □4 | Not at all |
| 88888 | **B16.5** | **Worrying thoughts go through my mind.** | |
|  |  | □1 | A great deal of the time |
|  |  | □2 | A lot of the time |
|  |  | □3 | Not too often |
|  |  | □4 | Very little |
| 88888 | **B16.6** | **I feel cheerful.** | |
|  |  | □1 | Never |
|  |  | □2 | Not often |
|  |  | □3 | Sometimes |
|  |  | □4 | Most of the time |
| 88888 | **B16.7** | **I can sit at ease and feel relaxed.** | |
|  |  | □1 | Definitely |
|  |  | □2 | Usually |
|  |  | □3 | Not often |
|  |  | □4 | Not at all |
| 88888 | **B16.8** | **I feel as if I am slowed down.** | |
|  |  | □1 | Nearly all the time |
|  |  | □2 | Very often |
|  |  | □3 | Sometimes |
|  |  | □4 | Not at all |

| 88888 | **B16.9** | **I get a sort of frightened feeling like ‘butterflies’ in the stomach.** | |
| --- | --- | --- | --- |
|  |  | □1 | Not at all |
|  |  | □2 | Occasionally |
|  |  | □3 | Quite often |
|  |  | □4 | Very often |
| Refuse to answer  88888 | **B16.10** | **I have lost interest in my appearance.** | |
|  |  | □1 | Definitely |
|  |  | □2 | I don’t take as much care as I should |
|  |  | □3 | I may not take quite as much care |
|  |  | □4 | I take just as much care as ever |
| 88888 | **B16.11** | **I feel restless as if I have to be on the move.** | |
|  |  | □1 | Very much indeed |
|  |  | □2 | Quite a lot |
|  |  | □3 | Not very much |
|  |  | □4 | Not at all |
| 88888 | **B16.12** | **I look forward with enjoyment to things.** | |
|  |  | □1 | As much as I ever did |
|  |  | □2 | Rather less than I used to |
|  |  | □3 | Definitely less than I used to |
|  |  | □4 | Hardly at all |
| 88888 | **B16.13** | **I get sudden feelings of panic.** | |
|  |  | □1 | Very often indeed |
|  |  | □2 | Quite often |
|  |  | □3 | Not very often |
|  |  | □4 | Not at all |
| 88888 | **B16.14** | **I can enjoy a good book or radio or television program.** | |
|  |  | □1 | Often |
|  |  | □2 | Sometimes |
|  |  | □3 | Not often |
|  |  | □4 | Very seldom |

Refuse to answer

88888

88888

88888

88888

88888

88888

88888

88888

88888

88888

88888

Refuse to answer

**SECTION I: DEMOGRAPHICS**

Refuse to answer

| **I1**  88888 | **What is your current occupation?** | | | | |
| --- | --- | --- | --- | --- | --- |
|  | □1 | Farmer | □6 | | Service- Government |
|  | □2 | Wage labourer | □7 | | Homemaker |
|  | □3 | Skilled worker | □8 | | Retired |
|  | □4 | Shop keeper | □9 | | Unemployed |
|  | □5 | Self-employed | □10 | | Others, please specify ________ |
| **I2**  88888 | **Were you working before you were seeking care at this hospital, for your illness?** | | | | |
|  | □1 | Working full-time | □3 | | Retired and not working |
|  | □2 | Working part-time | 4 | | Homemaker |
| **I3**  88888 | **How many persons live in your household?**  Number of people | | | | |
| **I4**  88888 | **How would you rate the economic status of your household?** | | | | |
|  | □1 | Poor | □3 | Upper Middle Class | |
|  | □2 | Lower Middle Class | □4 | Wealthy | |

**Thank you for completing the survey.**

1. Items in B1 are from the Functional Assessment of Chronic Illness Therapy system of Quality of Life questionnaires (“FACIT system”). The FACIT and all related works are owned and copyrighted by, and the intellectual property of David Cella, Ph.D. Permission for use of the FACT/FACIT system of questionnaire is obtained by contacting information@facit.org. [↑](#footnote-ref-1)
2. Items B13.1–B13.6 are from FACIT-Pal (Version 4) and B13.8–B13.9 are from FACT-G (Version 4). Both FACIT-Pal and FACT-G are from the Functional Assessment of Chronic Illness Therapy system of Quality of Life questionnaires (“FACIT system”). The FACIT and all related works are owned and copyrighted by, and the intellectual property of David Cella, Ph.D. Permission for use of the FACT/FACIT system of questionnaire is obtained by contacting information@facit.org. [↑](#footnote-ref-2)
3. Items B16.1-B16.14 taken from the Hospital Anxiety Depression Scale owned by R.P. Snaith and A.S. Zigmond. [↑](#footnote-ref-3)
